# Unique Coexistence of Two Resistive Switching Modes in a Memristor Device Enables Multifunctional Neuromorphic Computing Properties

**DOI:** 10.1021/acsami.4c07820

**Published:** 2024-08-12

**Authors:** Ayoub H. Jaafar, Salim Khalfan Suroor Al Habsi, Thomas Braben, Craig Venables, Maria Grazia Francesconi, Graeme J. Stasiuk, Neil T. Kemp

**Affiliations:** † School of Physics and Astronomy, University of Nottingham, Nottingham NG7 2RD, U.K.; ‡ Department of Chemistry and Biochemistry, University of Hull, Hull HU6 7RX, U.K.; § Department of Imaging Chemistry and Biology, School of Biomedical Engineering and Imaging Sciences, King’s College London, London SE1 7EH, U.K.

**Keywords:** memristor, volatile, nonvolatile, synapse, GeO_2_ nanoparticles, polymer

## Abstract

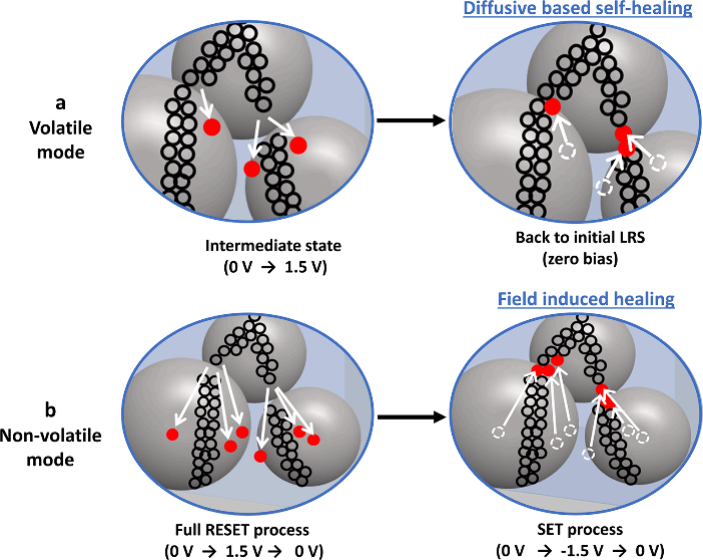

We report on hybrid memristor devices consisting of germanium dioxide nanoparticles (GeO_2_ NP) embedded within a poly(methyl methacrylate) (PMMA) thin film. Besides exhibiting forming-free resistive switching and an uncommon “ON” state in pristine conditions, the hybrid (nanocomposite) devices demonstrate a unique form of mixed-mode switching. The observed stopping voltage-dependent switching enables state-of-the-art bifunctional synaptic behavior with short-term (volatile/temporal) and long-term (nonvolatile/nontemporal) modes that are switchable depending on the stopping voltage applied. The short-term memory mode device is demonstrated to further emulate important synaptic functions such as short-term potentiation (STP), short-term depression (STD), paired-pulse facilitation (PPF), post-tetanic potentiation (PTP), spike-voltage-dependent plasticity (SVDP), spike-duration-dependent plasticity (SDDP), and, more importantly, the “learning–forgetting–rehearsal” behavior. The long-term memory mode gives additional long-term potentiation (LTP) and long-term depression (LTD) characteristics for long-term plasticity applications. The work shows a unique coexistence of the two resistive switching modes, providing greater flexibility in device design for future adaptive and reconfigurable neuromorphic computing systems at the hardware level.

## 1 Introduction

**Figure 1:**
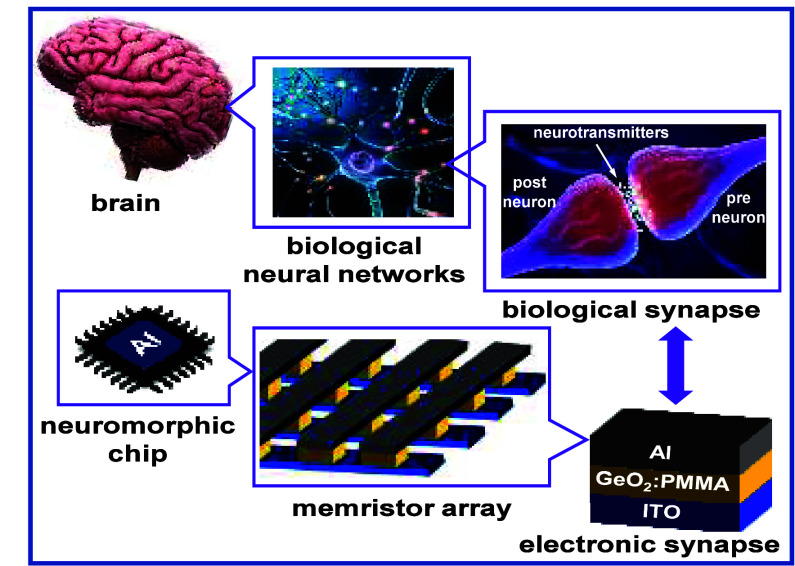
Neuromorphic computing chips are inspired by the human brain, mimicking its structure and function. The brain’s cortex contains roughly 100 billion neurons interconnected by about 100,000 billion synapses, enabling parallel information processing. Neurons, comprising dendrites, somas (main body within the nucleus for protein production and maintenance), and axons for small signal transmission, connect at synapses via small gaps (∼20 nm) where neurotransmitter levels adjust electrical conductance, influencing signal strength and synaptic plasticity through potentiation or depression. Memristors, serving as electronic arrays, offer a platform for spike-based learning and parallel neural computation, mirroring the brain’s complex learning capabilities. Reproduced or adapted with permission from iStock.com (SciePro/iLexx/libre des droit).

In addition to high-density data storage applications, memristors are showing increased promise for use in neuromorphic computing, where there is a growing need to overcome the bottleneck limitations of traditional von Neumann-based computing architectures and the scaling difficulties of “Moore’s law computing”, which is heavily reliant on increasing computational power by squeezing more and more transistors per unit area on a computer chip.^1−4^ Memristors offer excellent advantages, such as a simple 2-terminal device architecture,^5^ high density integration using a crossbar design,^6^ low voltage operation (less than 1.0 V),^7^ high write/erase speeds (pulse duration of 10 ns),^8^ long retention time (up to 10 years),^9^ high endurance (10^12^ cycles),^10^ and simple fabrication using a metal–insulator–metal (MIM) structure (where I is replaced with the active switching medium, typically an oxide or mobile metallic species).^11−16^ Inspired by key features of function and the learning properties of the human brain, memristors can be implemented in crossbar arrays and used in neuromorphic chips to mimic the analogue switching and learning characteristics of biological synapses for artificial intelligence applications (see Figure 1).^17,18^

Depending on their memory characteristics, memristors can be classified into two modes: long-term (nonvolatile or nontemporal) memory and short-term (volatile or temporal) memory. The coexistence of both modes in a single memristor offers a dynamic approach to modify the retention time of stored data by freeing up data that is stored for different purposes. For example, with long-term memory, the data can be stored for a long time before being erased, and this memory has been used for constructing feedforward neural networks (FNNs) for nonvolatile data processing applications.^19,20^ In the short term memory case, the data can be retained for a reasonable amount of time before they spontaneously erases. This method conserves system energy and time by eliminating the need for manual data erasure and has been employed in the processing of temporal data in a biofaithful fashion in recurrent neural network (RNNs).^21^ This type of memory can also be used as selector devices, which are placed in series with memory elements to solve the sneak-path issue in crossbar or multilayered constructions.^22,23^ Their volatile nature means they can also be used as reservoir elements in physical reservoir computing (RC) systems.^4,24−29^

In this article, we report the first example of a hybrid nanocomposite device that shows both volatile and nonvolatile modes that are programmable by controlling the stopping voltage. The coexistence of both modes have been reported in a few metal oxide based devices^4,30−32^ and an organic material of ferritin.^33^ In all of these reported devices, both modes are attained by regulating the compliance current (CC) level, e.g., volatile operation at low CC level and nonvolatile operation at high CC level. In contrast, the CC control was not needed for devices reported in this work.

In general, memristors (as fabricated devices) are initially in the HRS, which in most cases requires a forming process to activate devices for repeatable memristor switching.^34−36^ The forming process involves the application of high voltages (e.g., 6 V is needed to initiate a device consisting of 10 nm thick of tantalum oxide sandwiched between TiN electrodes).^35^ This process generates local defects or conductive nanofilament(s) across the device, which permit current to flow between the two electrodes. Forming processes are not desirable because they cause chemical and thermal damage of the switching layer, which reduces device yield and leads to device-to-device variability in the SET/RESET voltages.^37,38^ In addition, it adds complexity for learning processes and programming the algorithms of artificial neural systems, which typically operate at LRS.^37,39−41^ Several approaches have been applied to fabricate forming-free (as fabricated) memristors. These include doping of the switching matrix upon fabrication,^42,43^ introducing highly mobile ions into the switching matrix by deposition of active metal electrodes (Cu or Ag),^38^ and reducing the switching matrix thickness.^44^ Although these other devices are formation-free, they are not initially in the LRS. A forming-free memristor with initial LRS based on hybrid organic–inorganic materials, to the best of our knowledge, has not been reported.

Conventionally, memristors based on oxides are fabricated via complex and expensive techniques such as atomic layer deposition, sputtering, and chemical and physical vapor deposition techniques,^1,25,28,30,45,46^ which makes fabrication costs high and device fabrication slow. The high temperatures involved also prevent their use in flexible electronics. Hybrid organic–inorganic materials-based memristors can instead be fabricated via solution processing techniques, which offer advantages of low cost, vacuum free, and fast and large-area fabrication on lightweight and flexible substrates.^13,47−51^ Additionally, the solution processing technique, e.g., spin coating, supports massive array scalability as it has recently been used to realize a wafer-scale integration of high-density and reliable memristor arrays.^52^ Hybrid materials-based memristors have demonstrated not only excellent switching properties such as low-operation SET/RESET voltages, low power consumption, large resistance off/on ratios, and multilevel data storage capabilities but also suitability to a number of flexible neuromorphic applications.^47,50,53−56^ Furthermore, instead of employing inert matrix materials, polymers responsive to light, pressure, heat, chemicals, or gas can enhance memristors with additional functionality. Such polymers expand the range of capabilities of memristive devices, e.g., optically tunable artificial synapses,^48,57,58^ and can be integrated within advanced in-memory computing architectures for the development of ultrafast multisensory systems.

In this study, the unique programmable volatile and nonvolatile switching modes of the hybrid nanocomposite device were utilized to demonstrate essential synaptic learning functionalities that are required in many neuromorphic computing architecture; these include short-term potentiation (STP), short-term depression (STD), long-term potentiation (LTP), long-term depression (LTD), paired-pulse facilitation (PPF), post-tetanic potentiation (PTP), spike-voltage-dependent plasticity (SVDP), spike-dependent dynamic plasticity (SDDP), and “learning–forgetting–rehearsal”. The work lays the foundation for developing simple and low-cost electronic synapses with dynamically tunable and voltage-dependent reconfigurable properties for future artificial intelligence applications.

## 2 Experimental Section

### 2.1 Synthesis and Material Characterization of GeO_2_ NPs

GeO_2_ NPs were prepared by a low-temperature solvothermal process. In a typical procedure, thioacetic acid (TAA) (0.494 g, 6.58 mmol) was dissolved in acetic acid (30 mL), and then the solution was transferred into a Teflon-lined vessel (50 mL). After the addition of germanium tetrachloride (0.15 mL, 1.32 mmol), the vessel was sealed in a stainless-steel autoclave and heated to 140 °C for 24 h. Subsequently, the autoclave was naturally cooled to ambient temperature. The off-white precipitate was collected by filtration and washed with ethanol, carbon tetrachloride, concentrated hydrochloric acid, and water several times. Finally, the resulting product was dried at 60 °C for 2 h. The prepared material was characterized by scanning electron microscopy (SEM), energy-dispersive spectroscopy (EDX), X-ray diffraction (XRD), and Raman spectroscopy.

### 2.2 Device Fabrication and Measurement

The mixed hybrid (nanocomposite) devices, consisting of a blended mix of GeO_2_ NPs and PMMA polymer with an ITO/GeO_2_:PMMA/Al architecture (see the inset in Figure 3a), were fabricated using the following procedure. ITO-coated glass (Delta Technologies), as a bottom electrode, was initially cleaned by sequential ultrasonic cleaning in acetone, propan-1-ol, and deionized water (DI) for a duration of 5 min each, followed by a final rinse with DI water and dried by N_2_ flow. The GeO_2_ NPs in toluene (3%) were dispersed in a PMMA (*M*_w_ = 120,000, Sigma-Aldrich)/toluene solution (5%) and spin-coated (1000 rpm, 30 s) onto the ITO substrate to form a thin film of ∼200 nm thickness. Annealing at 100 °C for 15 min in air was used to remove residual solvent. The mass fraction (*R*_m_) of the GeO_2_ NPs blended in PMMA was 0.05% and 0.15%. We expect the NP distribution to be fairly uniform within the polymer but with occasional clusters as shown by atomic force microcopy (see Figure S2). A control device containing only a PMMA thin film (200 nm) was also formed. For both device types, final Al films (200 nm) were thermally deposited onto the substrates using a shadow mask with 400 μm diameter circular dots to act as the top electrodes. Note that besides the hybrid devices, a single-layer device consisting of only GeO_2_ NPs and a bilayer device with distinct layers of the GeO_2_ NPs and PMMA were also investigated. Their fabrication method and electrical characteristics are presented in the Supporting Information. A probe station equipped with a Keithley 4200A-SCS semiconductor parameter analyzer unit was used to determine the electrical characteristics.

## 3 Results and Discussion

### 3.1 GeO_2_ NPs Material Characterization

**Figure 2:**
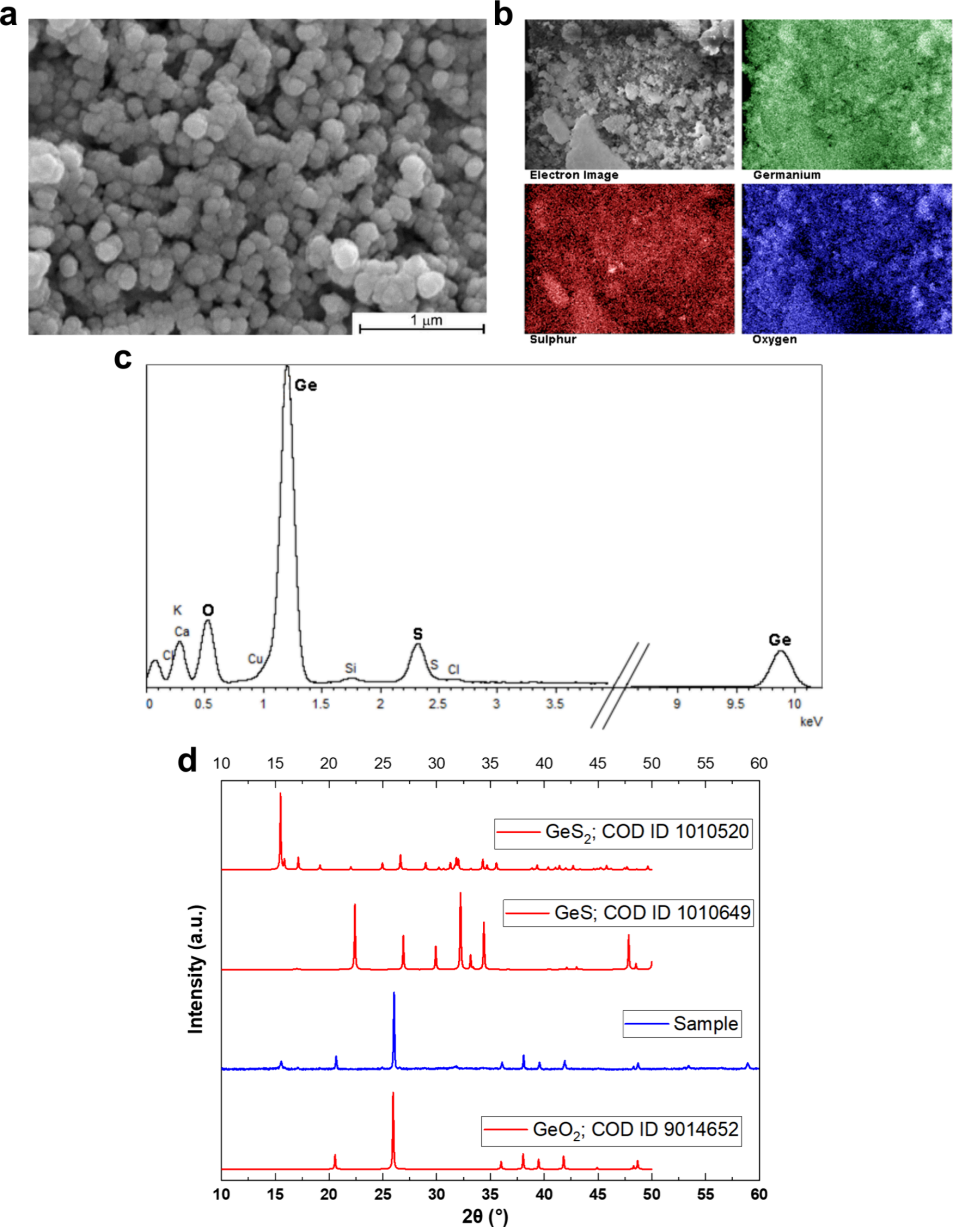
(a) SEM image of the GeO_2_ NPs showing diameters of 100–200 nm. (b) EDX color mapping showing chemical constituents of germanium, sulfur, and oxygen. (c) EDX spectrum of the GeO_2_ NPs showing signature peaks of germanium, oxygen, and sulfur. (d) XRD spectrum of the GeO_2_ NPs (blue) in contrast to known GeS_2_, GeS, and GeO_2_ spectra.

The morphology and composition of the GeO_2_ NPs were investigated using SEM, EDX, XRD, and Raman. Figure 2a shows an SEM image of the as-fabricated quasi-spherical GeO_2_ NPs material with diameters of 100–200 nm. EDX maps and spectra were taken to investigate the chemical composition of the material and are shown in Figures 2b and 2c, respectively, showing Ge, O, and S elements in the sample area, with a Ge:O ratio of 1:2.3, Ge:S 1:0.51, and O:S 1:0.22. X-ray diffraction (XRD) measurements were performed to elucidate the structure of the material, as shown in Figure 2d. The XRD confirms the GeO_2_ material with characteristic peaks of 2θ at 20°, 26°, 36° 39°, and 42°, which is consistent with an GeO_2_ reference. We show the GeS and GeS_2_ references to indicate that the sulfur is not incorporated into the nanomaterial lattice, thus suggesting that the TAA acts as a capping agent and the material is dominantly a GeO_2_ nanomaterial. The room temperature Raman spectrum of the GeO_2_ NPs material deposited on ITO-coated glass is shown in Figure S1.

### 3.2 Electrical Properties

**Figure 3:**
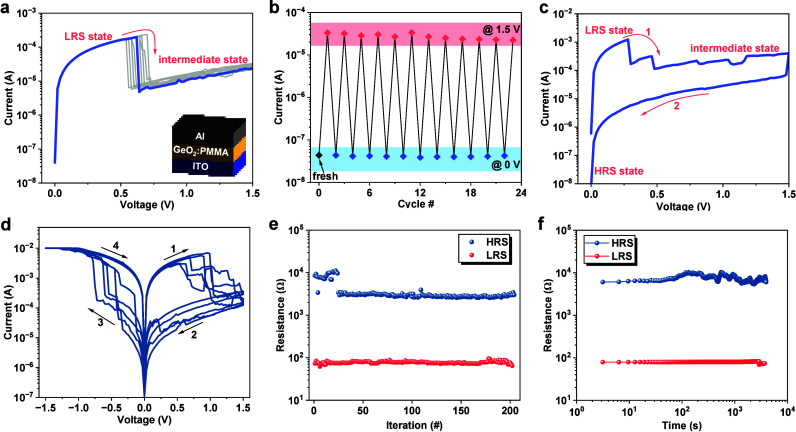
Stopping voltage-dependent switching modes. (a) Consecutive *I–V* curves for a device consisting of GeO_2_ NPs embedded within PMMA at *R*_m_ = 0.05% showing short-term memory effect with a stopping voltage of 1.5 V. The inset shows a schematic of the hybrid memristor device architecture. (b) Switching between the LRS and volatile intermediate state. (c) A full *I–V* sweep (i.e., stopping voltage of 0 V) with direction of 0 V → 1.5 V → 0 V. (d) Consecutive *I–V* curves upon sweeping the device between ±1.5 V, showing the nonvolatile bipolar memory switching. (e) Programming endurance test of a hybrid GeO_2_ NPs:PMMA device for more than 200 cycles. (f) Retention test characteristics for the HRS and LRS up to 3900 s.

The devices are unique in that they exist in the LRS state in their initial condition, and they exhibit both volatile (short-term memory) and nonvolatile (long-term memory) switching modes upon controlling the applied stopping voltages. Figure 3 shows both voltage-dependent switching modes of the device with GeO_2_ NPs concentration of *R*_m_ = 0.05% at different stopping voltages. The *I–V* curve of the device at the stopping voltage of 1.5 V is illustrated in Figure 3a. Upon sweeping from 0 to 0.62 V the device switches from the initial LRS to the HRS, staying in this state until 1.5 V (stopping voltage). After a short time (see Figure S5e for relaxation time) with no applied voltage, the device reverts to its initial state. Subsequent *I–V* sweeps (gray curves) show repeatability of the volatile switching mode. This mode can be clearly observed in Figure 3b, which plots the repeated cycles between 0 and 1.5 V. In contrast, a full voltage sweep from 0 to 1.5 V and back to 0 V (stopping voltage) switches the device to the HRS (see Figure 3c). In this scenario, only a sweep to negative voltage polarity could revert the device back to its initial LRS. This resulted in the nonvolatile (bipolar switching memory) mode as shown in Figure 3d. It is worthwhile to note that both the volatile and nonvolatile switching modes work with either positive or negative polarities, as shown in Figure S3 and seen in other hybrid system and oxide thin films.^59,60^ Reproducibility of the volatile and nonvolatile modes was observed in a range of devices, albeit with some cycle-to-cycle variability (Figure S6) and device-to-device variability for the volatile (Figure S4a) and nonvolatile modes (Figure S4b).

To evaluate the RS stability of the hybrid memristor, cycling endurance characteristics for HRS and LRS were performed. The device showed a highly stable and reproducible operation up to 200 cycles with an ON/OFF ratio of about 2 orders of magnitude without any degradation, as shown in Figure 3e. The endurance characteristics were achieved by resetting/setting the device by voltage pulses of −1.5 V/1.5 V for 100 ms with a read voltage of 0.05 V. Furthermore, the stability of the HRS and LRS over time was also examined. Figure 3f shows the data retention test for both states. Following 3900 s, the values of both states were virtually unaltered, and the ON/OFF ratio was maintained, indicating that the device has a good reliability and RS uniformity.

The variability of the initial conductance of the hybrid ITO/GeO_2_:PMMA/Al devices was quantified by measuring the *I–V* properties across 20 devices and plotting the value of the resistances at 0.1 V using the Weibull percentiles, as shown in Figure S4c. The initial resistance of all the devices ranged between 10^2^ Ω and 10^3^ Ω, and their *I–V* properties had linear (ohmic) characteristics (Figure S4d), which confirmed the devices are in the LRS.^61,62^ The linear relationship between *I* and *V* means that the devices are initially in LRS, and their metallic-like conducting filaments are already formed in their pristine state.

**Figure 4:**
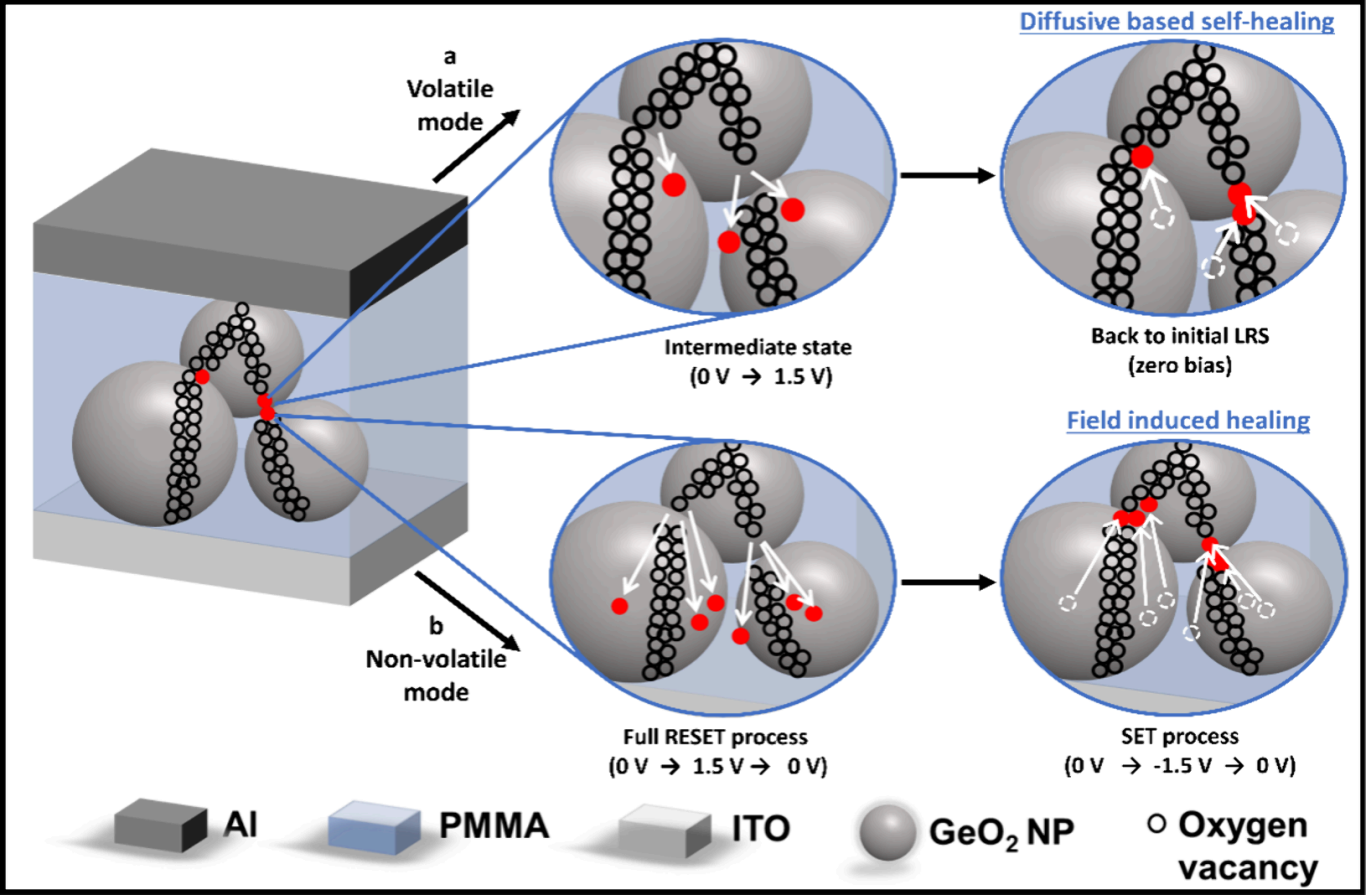
Schematic showing the resistive switching mechanism in the mixed hybrid memristor device GeO_2_ NP:PMMA (at *R*_m_ = 0.05%). The two pathways (arrows) show the two possible modes: (a) volatile mode (short-term memory) and (b) nonvolatile mode (long-term memory), which are obtained by applying different stopping voltages, Intermediate RESET (0 → +1.5 V) or Full SET process (0 → −1.5 → 0 V).

Based on the above electrical properties, the small Ge–O bond formation energy, and high defect density of oxygen vacancies in Ge–O at low temperatures,^63,64^ we propose that the switching mechanism of the volatile and nonvolatile modes is due to the rupture and reforming (either self-healing or electric-field-induced healing) of oxygen vacancy filament(s) within the GeO_2_ NPs. It is generally accepted that oxygen vacancies play a vital role in the switching mechanism of memristor devices.^65^ Oxygen vacancies can also act as a charge trapping sites, facilitating the switching effect in GeO_2_-based devices.^66^ The schematic illustrations of the switching mechanism for both volatile and nonvolatile modes, achieved by controlling the stopping voltage, are shown in Figure 4. The initial LRS and forming-free switching effect indicate that the devices in their pristine state already have conducting pathways (filaments) that bridge across the two electrodes. In nanoparticle/polymer composite systems, it is known that conducting pathways can occur when the volume fraction of NPs embedded in the electrically inert host is above a certain critical volume threshold, and this can occur in a homogeneous^67,68^ or inhomogeneous^57^ manner via percolative effects. In the mixed GeO:PMMA hybrid device, the NP diameter is large and similar to the electrode separation, so the critical volume fraction is certainly reached, and it is likely the current path is directly through a single (or few) NPs. Upon sweeping the device to higher voltages, typically at a RESET voltage of ∼0.6 V, there is a sudden switch to an intermediate HRS (see Figure 3a). We attribute this to a Joule heating effect, which causes a migration/diffusion of oxygen vacancies away from the filament at its weakest point (highest electrical resistance point of the filament heats proportionally more than other parts of the filament) and breaks the filament; see Figure 4 (a Volatile mode, Intermediate RESET state). Note that it has been recently observed that oxygen vacancies can migrate along the direction of the applied electric field and form a conducting channel between the two electrodes, as shown in a tungsten trioxide-based memristor via *in situ* X-ray multimodal imaging.^65^ Moreover, a spontaneous migration/diffusion of the oxygen vacancies away from the metal/semiconductor interface after removing the applied voltage, causing a volatile switching effect, has been also reported in a tungsten trioxide-based analogue memristor.^69^ After the filament is broken the device retains its resistance state up to the stopping voltage of 1.5 V. Subsequently, after the applied voltage is removed, the point contact of the conductive filament(s) is reformed again (self-heals), most likely through a thermal diffusion process;^70−72^ see Figure 4 (a Volatile mode, zero bias), switching the device back into its initial LRS. This spontaneous switching indicates a volatile (short-term) switching mode.

In the case of a full positive potential cycle, 0 → +1.5 → 0 V, the device switches from the LRS to HRS (RESET) due to the Joule heating effect, which causes a rupture of the filament(s) as before. However, on returning to 0 V, the device stays in the HRS state (Figure 3c). The switching is nonvolatile since the HRS is maintained after the applied voltage is removed. The cause of this is likely due to the additional time the device is subjected to a positive potential (because of the return back to 0 V), which enhances the migration of oxygen vacancies far away from the filament(s) and increases the damage in the broken filament to a point where thermal diffusion alone is insufficient to reform the connection; see Figure 4 (b Nonvolatile mode, RESET process). In contrast, a full cycle consisting of positive and negative potentials, 0 → +1.5 → −1.5 → 0 V, reconnects the filament and enables recovery of the LRS at −1.5 V (Figure 3d). The recovery of the LRS during the SET is likely due to the reversed direction of the field, which causes oxygen vacancies to move back toward the broken filament regions; see Figure 4b (b Nonvolatile mode, SET process). Similar nonvolatile switching behavior with an initial LRS has been demonstrated recently in memristors based on TiO_2_^61^ and MA_3_Sb_2_Br_9_ perovskite^62^ with a switching attributed to the rupture and formation of conductive filament. It is worth mentioning that the switching in our devices is not due to the electrochemical metallization (ECM) process^73^ since inert electrodes were used. In addition, the ECM process depends on the redox reactions, which are voltage polarity dependent, whereas the volatile and nonvolatile switching modes in our devices can occur upon applying either positive or negative polarities. Besides, memristors with the switching mechanism based on ECM process typically exhibit an initial HRS and a forming process is needed,^74^ which is not the case in this work. Lastly, we do not attribute the switching effects to changes in an interfacial Schottky barrier, since the switching from the LRS to the HRS is very sharp and like that seen in filamentary devices.

In general, forming processes are required for RS devices to initiate the devices for the RS cycling by forming conductive nanoscale (e.g., oxygen vacancies Vo) filaments within the switching matrix. This is normally achieved by applying voltages that are much higher than the SET voltage.^75,76^ The forming-free process observed in our devices can be attributed to the initially formed defects within the GeO NPs.^77,78^ The elimination of the forming process in our devices simplifies the device operation and is an advantage compared with many devices reported in the literature.

### 3.3 Neuromorphic Switching Properties

**Figure 5:**
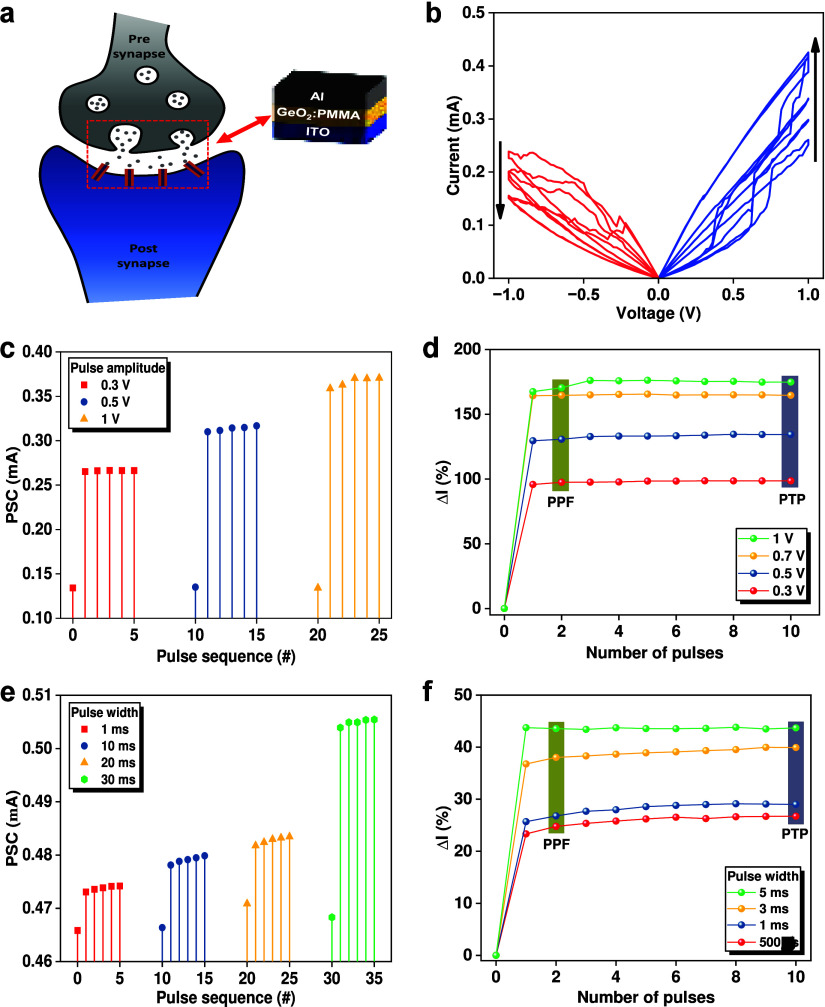
(a) Schematic representation of a biological synapse and a memristor device showing the correspondence between biological and electronic synapses. (b) Consecutive *I–V* characteristics showing the analogue switching behavior of the device. (c) Current response of the memristor for pulses with different amplitudes, emulating SVDP. Note: the PSC was read at 0.05 V. (d) Mean changes in the current during the application of 10 pulses for pulse trains with different pulse amplitudes. (e) Current response of the memristor for pulses with different pulse duration (width), emulating SDDP behavior. (f) Mean changes in the current during the application of 10 pulses for pulse trains with different pulse durations.

The potential use of the hybrid device as an electronic synapse (Figure 5a) for neuromorphic computing systems was investigated. In Hebbian learning, the connection strength (conductance) of a synapse can be modified through repeated stimuli. Figure 5b (red curves) shows that the conductance of the device can be gradually decreased by application of consecutive negative *I–V* sweeps with the same value of −1 V. The decrease of conductance indicates a progressive rupture of the conductive filament(s). After setting the device to the OFF state, a series of positive *I–V* sweeps to +1 V perform the opposite operation and gradually increases the conductance by reforming the conductive filament(s) (Figure 5b, blue curves). These results clearly indicate the neuromorphic switching capability of the device, whereby the conductance and synaptic plasticity of the state can be progressively controlled.^79^

The ability to modify the strength of synapses through the timing and voltage of spikes is crucial for learning and cognition in many parts of the human brain. This is especially relevant in the hippocampus, where voltage spikes are instrumental in driving synaptic plasticity processes such as LTP and LTD, which are essential for learning, memory formation/retrieval, and cognitive function. Figure 5c demonstrates spike-voltage-dependent plasticity (SVDP) in the artificial synapses (hybrid nanocomposite memristor) through the application of continuous pulse trains with different voltages (0.3, 0.5, and 1 V) (constant pulse duration of 1 ms and pulse interval of 10 ms). The increasing spike voltage increments the postsynaptic current (PSC) as the pulse amplitude increases from 0.3 to 1 V. The relative differences in the PSC, Δ*I*, calculated using the equation , are presented in Figure 5d.

Paired-pulse facilitation (PPF) and post-tetanic potentiation (PTP) facilitate changes in synaptic transmission through the application of closely spaced presynaptic action potentials. Paired-pulse facilitation involves two closely spaced action potentials, whereas post-tetanic potentiation involves a period of high-frequency stimulation. Figures 5e and 5f demonstrate spike-duration-dependent plasticity (SDDP) behavior, in which the strength of the connection increases by increasing the pulse duration from 1 to 30 ms (fixed amplitude at 0.5 V and interval at 1 ms). It is expected that increasing the pulse duration forms a larger and more stable filament, leading to an increased PSC. The effect of varying the interval pulses was also studied and is shown in Figure S5a, with cycle-to-cycle and device-to-device variation for the pulse trains shown in Figures S5b and S5c, respectively.

The temporal dynamics of the volatile distance state after a single voltage pulse was also investigated. Figure S5d shows an increase in the PSC (read at 0.05 V) after the application of a single pulse with a duration of 1 ms at 0.5 V. Once the pulse is removed, the device spontaneously relaxes into an intermediate state. This sharp transition represents a short relaxation time and was found to be 200 ms based on the stretched-exponential based function model^80^ (see Figure S5e).

**Figure 6:**
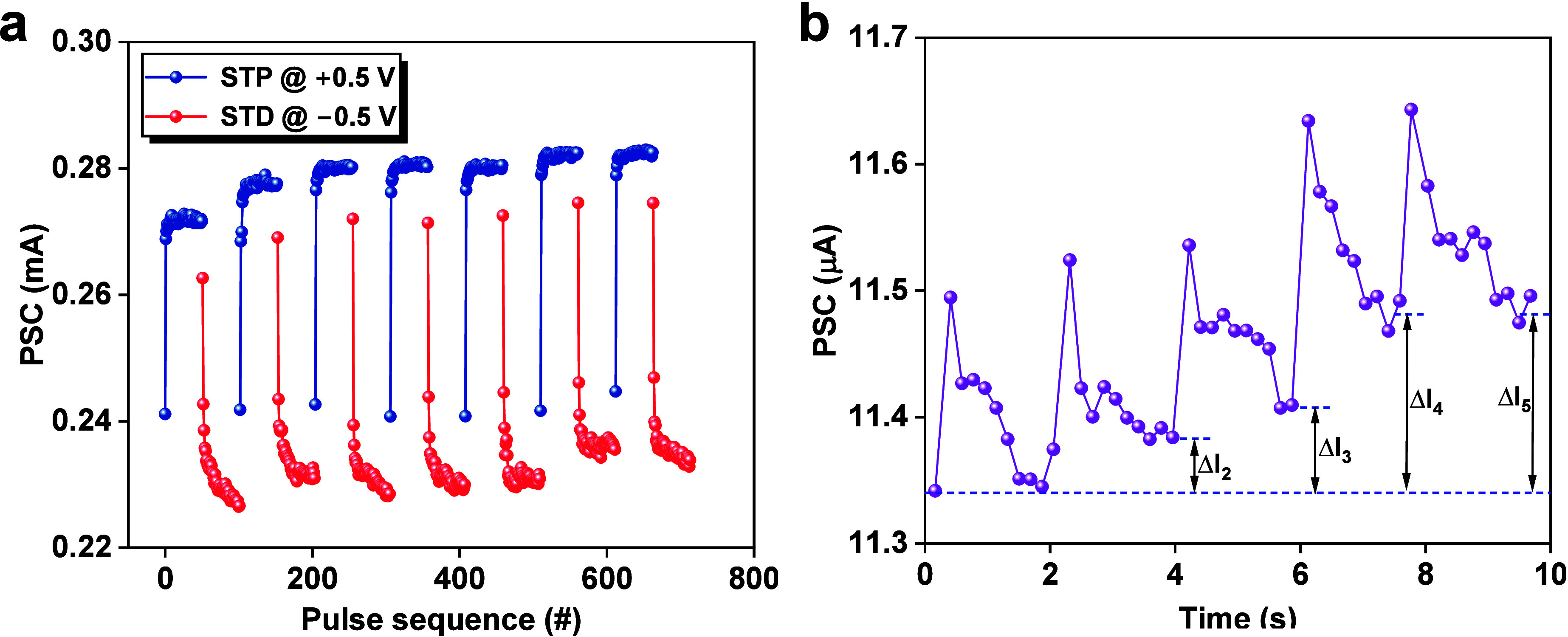
(a) PSC changes with a series of positive voltage pulses (0.5 V, 1 ms) followed by a series of negative voltage pulses (−0.5 V, 1 ms), showing the STP and STD. The PSC was read at 0.05 V. (b) Learning–forgetting–rehearsal behavior of five cycles. The PSC was read at 0.05 V.

Utilizing voltage spikes for both spike timing potentiation (STP) and spike timing depression (SDP) is hugely important for the temporal processing of information in neuromorphic applications. Figure 6a demonstrates STP (blue data) and STD (red data) effects through the application of 50 rapid successive identical positive pulses (0.5 V, 1 ms), followed by 50 successive identical negative pulses (−0.5 V, 1 ms). The effect is repeatable for at least 7 cycles. The STP and STD suggest the temporal dynamic of synaptic weight that lasts only a few milliseconds.^81^

Synaptic consolidation is important for the reinforcement of memories and the conversion of short-term memory (STM) to long-term memory (LTM). Figure 6b demonstrates such a process through repeated application of a pulse train. Here, a more advanced synaptic function, involving changes in the synaptic plasticity, called “learning–forgetting–rehearsal” behavior, is shown. Such a behavior is explained by the Ebbinghaus forgetting curve where the memory is strengthened through repetitive learning.^17,82^Figure 6b shows that the LTM process is achieved by the application of a single (learning) pulse train (2 V, 30 ms) followed by the absence of pulses (relaxation) for the forgetting process. After repeating the learning and forgetting processes for five cycles, the conductance of the electronic synapse increases (Δ*I*_5_ > Δ*I*_4_ > Δ*I*_3_ > Δ*I*_2_), indicating that relearning the previously stored information can strengthen the memory ability. In contrast, the devices having nonvolatile resistive switching mode exhibited a LTP and LTD, as shown in Figure S5f.

## 4 Conclusions

In summary, the work demonstrates a simple and low-cost solution processing strategy for the fabrication of hybrid GeO_2_ NP:PMMA memristors with a unique set of device properties including forming-free operation, LRS to HRS switching characteristics, and a novel stopping voltage-dependent switching behavior, which permits selectable device operation in either short-term memory (volatile) or long-term memory (nonvolatile) modes. Importantly, these modes can be programmed dynamically and interchangeably with modulation via low voltage operations. A spectrum of synaptic behaviors, both analogue and spiking-dependent, were exhibited, underscoring the device’s significance as a programmable synapse in neuromorphic computing. These behaviors encompassed short-term potentiation (STP), short-term depression (STD), long-term potentiation (LTP), long-term depression (LTD), paired-pulse facilitation (PPF), post-tetanic potentiation (PTP), spike-voltage-dependent plasticity (SVDP), spike-dependent dynamic plasticity (SDDP), and “learning–forgetting–rehearsal”. While these results demonstrate proof of concept of the new materials synthesis and device fabrication approach, further optimization will be needed to control and reduce the size of the nanoparticles for improved device scaling. A deeper understanding of the role of chemical and electronic defects in the material should also lead to improved device-to-device variability. Nevertheless, these findings suggest that the hybrid GeO_2_:PMMA material holds significant potential as a key component in new dynamically reconfigurable and adaptive artificial synapses, paving the way for future advances in smarter, more energy efficient neuromorphic computing systems.

## Supporting Information Available

Raman spectroscopy of the GeO_2_ NP material; AFM imaging of the surface of a GeO_2_ NP:PMMA hybrid device showing the nanoparticles distributed within the PMMA polymer; stopping voltage-dependent switching modes under negative polarity conditions; variability of the LRS state for the hybrid memristor; various current responses under different voltage pulsing schemes and showing cycle-to-cycle and device-to-device variability as well as relaxation times of the volatile state; endurance testing of a hybrid device using successive *I*–*V* curves; tests on devices with different NP concentrations and fits to the SCLC model; cumulative probability plots of the device endurance for low and high NP concentration devices; device characteristics for single-layer GeO_2_ and bilayer (GeO_2_/PMMA) devices, energy level diagram for a bilayer device (PDF)
