# Supplementary material for: Unique Coexistence of Two Resistive Switching Modes in a Memristor Device Enables Multifunctional Neuromorphic Computing Properties
Source: ACS Appl Mater Interfaces. 2024 Aug 12;16(33):43816–26. doi: 10.1021/acsami.4c07820 (PMC11345731; doi:10.1021/acsami.4c07820)
Supplement: Supplementary file 1 — am4c07820_si_001.pdf [file am4c07820_si_001.pdf]

Unique Co-existence of Two Resistive Switching Modes in a Memristor Device Enables Multifunctional Neuromorphic Computing Properties

Ayoub H Jaafar,[a,] Salim Khalfan Suroor Al Habsi,[b] Thomas Braben,[a] Craig Venables,[a] Maria Grazia Francesconi,[b] Graeme J Stasiuk,[c] and Neil T Kemp[a,*]*

[a] School of Physics and Astronomy, University of Nottingham, Nottingham, NG7 2RD, U.K.

[b] Department of Chemistry and Biochemistry, University of Hull, Hull HU6 7RX, U.K.

[c] Department of Imaging Chemistry and Biology, School of Biomedical Engineering and Imaging Sciences, King's College London, London SE1 7EH, U.K.

*Author to whom correspondence should be addressed. Electronic mail:

ayoub.hamdiyah@nottingham.ac.uk, neil.kemp@nottingham.ac.uk

Supporting Information

1. Raman Spectroscopy for GeO_2 NP Material

Room temperature Raman analysis using HORIBA LabRAM HR Raman Microscope, with multiple excitation wavelengths of 532 nm, 660 nm, and 785 nm was conducted to investigate the GeO_2 NP material. Figure S1 shows the Raman spectra of the GeO_2 NPs material deposited on ITO coated glass. The Raman spectra (smoothed using a Fast Fourier Transform filter in OriginLab) was largely dominated by the glass substrate signal. Consequently, the focus is on the peaks relevant to the GeO_2 NP material. All three excitation wavelengths show a peak centered at 445 cm^{-1} which is attributed to the α-quartz GeO_2[1,2] and the symmetric stretching vibration of the Ge-O-Ge bonds. However, for the 532 nm excitation wavelength, a peak centered at 344 cm^{-1} was observed and it can be related to bond-bending modes and the A_1-type bond-stretching of Ge-S bending of glassy-GeS_2, as reported in previous studies.[3,4] Note, observation of this peak in the 532 nm spectra and not the other excitation wavelengths is due to the photon-energy dependence of Raman spectra of GeS_2.[3] Some presence of GeS_2 within the GeO_2 is clear from the Raman spectra, however the EDX data in Figure 2 indicates a larger prevalence of GeO_2 within the material. In general, EDX is considered more quantitative than Raman spectroscopy, so we expect that GeO_2 makes up the much larger contribution to the material. Since the movement of oxygen vacancies dominates the switching mechanism across a wide range of metal oxide

materials, we infer that the switching mechanism in the material is because of the GeO_2 rather than the GeS_2.

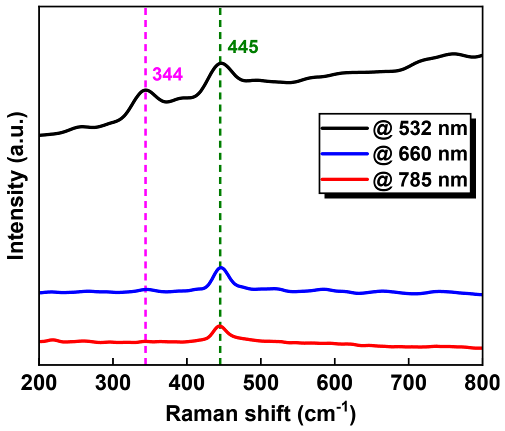

Figure S1. Raman spectra of the GeO_2 NPs prepared by a low temperature solvothermal process and showing peaks at 445 cm^{-1} (attributable to α-quartz GeO_2) and 344 cm^{-1} (attributable to GeS_2).

2. Mixed Hybrid GeO2 NP:PMMA Devices

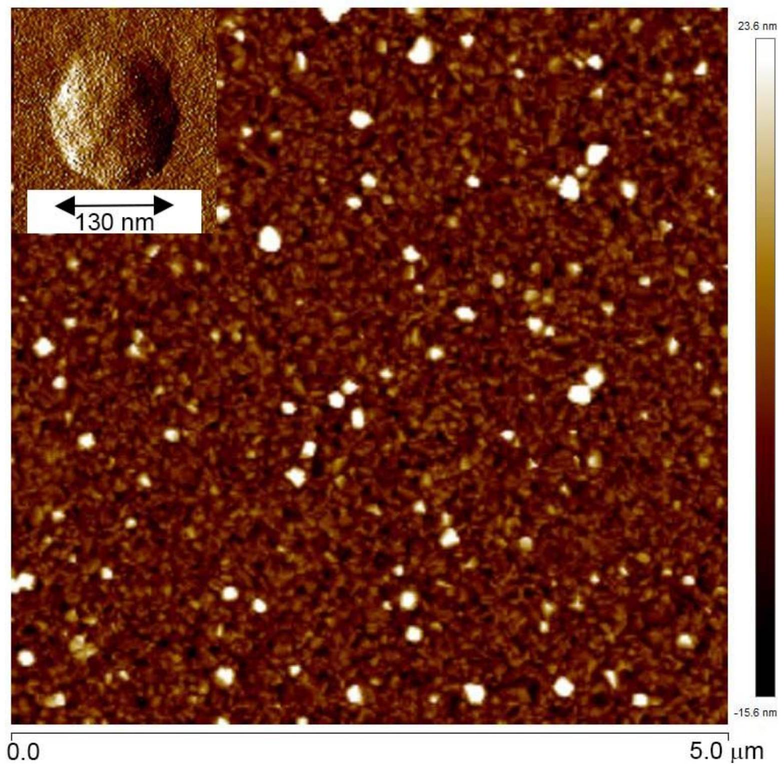

Figure S2. Atomic Force microscopy (AFM) of a GeO2 NP:PMMA hybrid device showing the top surface morphology of the device and nanoparticles distributed within the PMMA polymer.

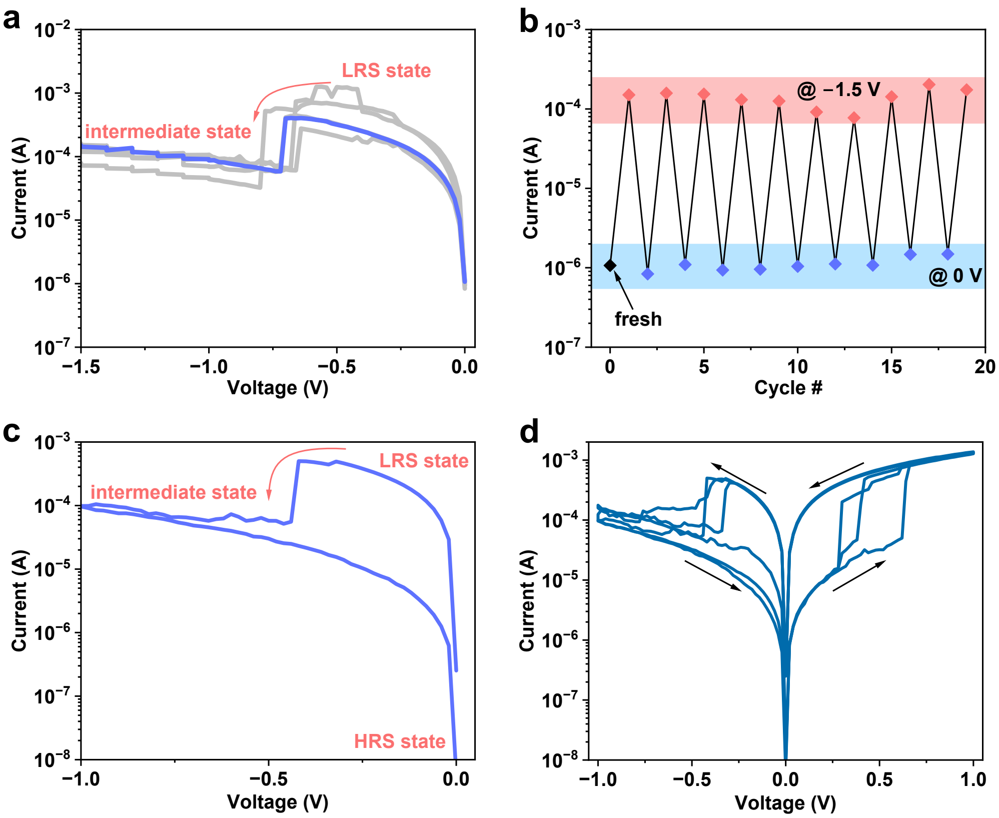

Figure S3. Stopping voltage-dependent switching modes under negative polarity conditions. a) Consecutive *I*-*V* curves for a device consisting of GeO_2 NPs embedded within PMMA at R_m=0.05% showing a short-term memory at a stopping voltage of −1.5 V. b) The switching between LRS and intermediate state upon repeated cycling between 0 V and −1.5 V. c) *I*-*V* curve at the stopping voltage of 0 V (in a voltage direction of 0 V → −1.0 V → 0 V). d) Consecutive *I*-*V* curves upon sweeping the device between ±1.0 V, showing the non-volatile bipolar memory switching.

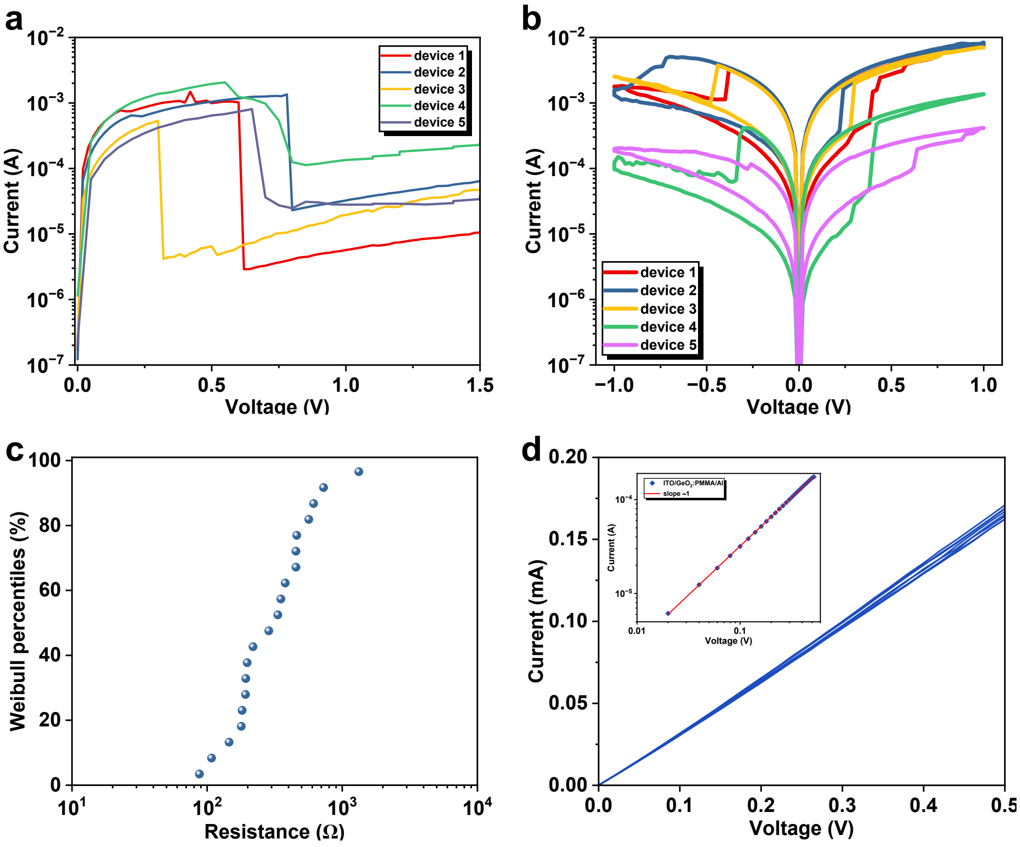

Figure S4. Variability of the LRS in forming-free hybrid GeO_2:PMMA-based memristors. In fact, since the NP concentration in the polymer is small (Rm = 0.05%) each device could have a variation in NP loading, thus resulting in a variation in the LRS levels. a) *I*-*V* sweeps taken from five devices, showing the volatile mode. b) *I*-*V* sweeps taken from five devices, showing the non-volatile bipolar RS memory. c) Weibull percentiles versus initial resistance of 20 devices. d) *I*-*V* sweeps for 10 initial LRS memristors in linear scale. The inset shows a fitting for an *I*-*V* sweep in the log I – log V scale. The linear relationship between I and V with a slope of 1 indicates the filaments are already formed between the electrodes and the device is initially in the LRS.

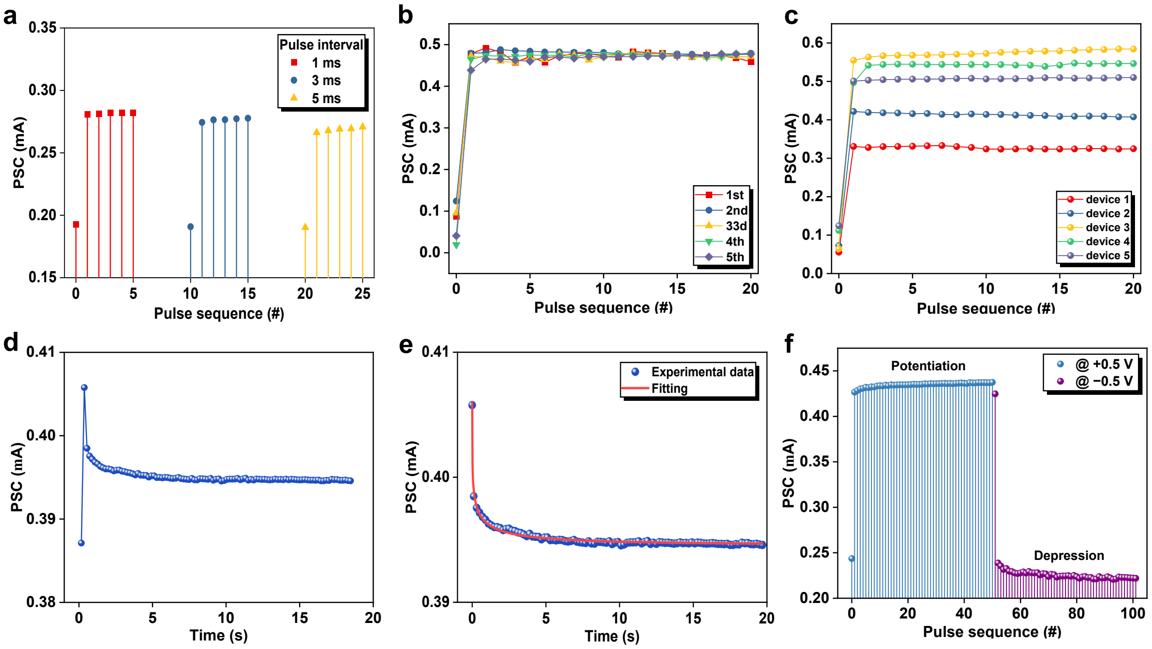

Figure S5. a) Current response of the hybrid GeO_2:PMMA device at Rm = 0.05% for pulses with different pulse intervals at fixed pulse duration of 1 ms and amplitude of 0.5 V, demonstrating spike-rate-dependent plasticity (SRDP). b) Cycle-to-cycle response of a device upon applying 20 pulses (pulse duration of 30 ms, pulse interval of 100 μs @ 1 V). The plot shows that the device exhibited a uniform cycle-to-cycle performance. c) Device-to-device variability upon applying 20 pulses (pulse duration of 30 ms, pulse interval of 100 μs @ 1 V). The variation in the PSC levels is likely related to the variation in the NP distribution across the sample. d) Relaxation time recorded after applying a single pulse with a duration of 1 ms at 0.5 V. e) A stretched-exponential based function (SEF) fit. The SEF was used to evaluate the relaxation time of the volatile LRS state. The current level is modelled by an exponential equation $I(t) = I_0\, exp[-({}^{t}/_{\tau})^{\beta}]$. Here $I(t)$ is the memory (current) level at a given time t, I_0 is the memory level at t = 0, τ is the characteristic relaxation time, which can be used to evaluate the forgetting rate. β is the stretch

index and was fitted to be 0.2 in this work. The fitted relaxation time was found to be 200 ms. f) long-term potentiation and long-term depression under 50 positive pulses (0.5 V, 3 ms) and 50 negative pulses (−0.5 V, 3 ms), respectively.

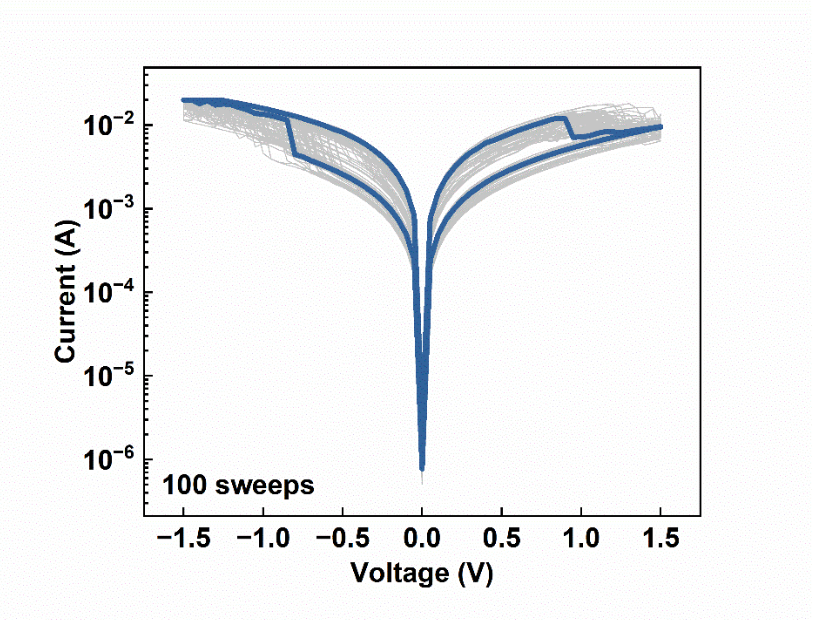

Figure S6. Successive *I*-*V* curves of a typical hybrid device, ITO/GeO_2:PMMA/Al (Rm = 0.05%), showing good reproducible switching performance for 100 sweeps in the non-volatile mode.

In Figure S7, the effect of NP concentration on the *I*-*V* characteristics, the OFF/ON ratio and the conduction mechanism was studied. As can be seen from Figure S7a, the device with R_m = 0.05% (blue curve) shows a typical *I*-*V* bipolar resistive switching behavior where the switching between HRS and LRS and vice versa occurs at different voltage polarities. It is well known that the PMMA is a good insulator and has no RS properties,[5] (see Figure S7a, red curve) and hence, the resistive switching properties of the devices are dominated by GeO_2 NPs. Importantly, most of the pristine devices were originally in LRS. During the sweep from 0 V to 1.5 V, the current

decreased with a particularly large transition at about 1 V, denoting the switch from the LRS to the HRS. In contrast, upon sweeping from 0 to –1.5 V, the current increased with a sharp transition at –0.8 V, denoting the switch back to the LRS. The device has a large resistance OFF/ON ratio of larger than two orders of magnitude. The asymmetric shape of the *I*-*V* curve can be ascribed to the different work functions of the ITO and Al electrodes, as has been shown by others[6,7] Note, a few devices were initially in the HRS, which can be attributed to the inhomogeneous distribution of GeO_2 NPs in the PMMA across the substrate. Upon increasing the NP concentration to Rm = 0.15%, the devices exhibited smooth bipolar transitions between HRS and LRS with a very small OFF/ON resistance ratio and had the highest conductance (see Figure S7a, yellow curve). The effect of GeO_2 NPs on the RS properties can be clearly seen in Figure S7b, which plots the ON and OFF currents and the OFF/ON resistance ratio as a function of the NP concentration.

To investigate the conduction mechanisms in the mixed hybrid device, ITO/GeO_2:PMMA/Al, the *I*-*V* characteristics of devices at R_m= 0.05% and 0.15% were replotted on a log I – log V scale, as shown in Figure S7c and S7d, respectively. For the devices with R_m= 0.05%, ohmic and SCLC transport dominate, whereas for the higher concentration, R_m= 0.15%, only ohmic conduction is observed.

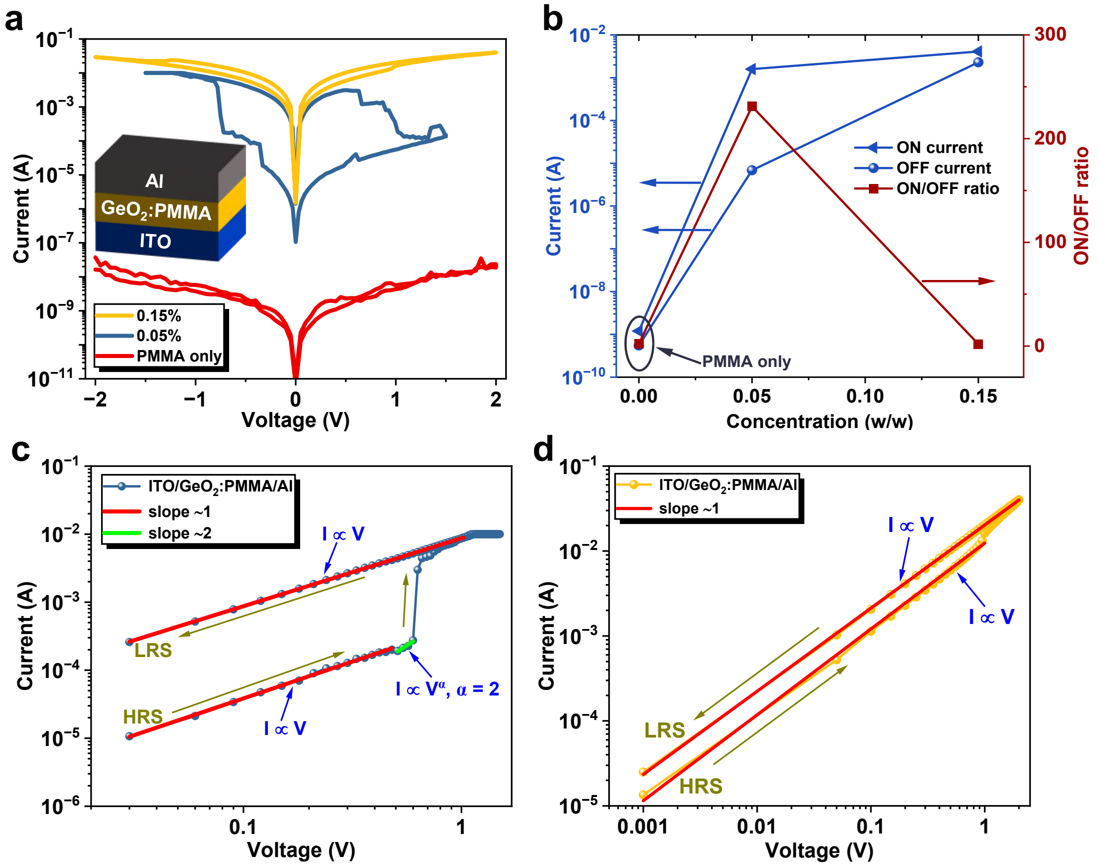

Figure S7. a) *I*- *V* sweep for a device consists of GeO_2 NPs embedded within PMMA at R_m=0.15% (yellow curve), R_m=0.05% (blue curve) and PMMA only (red curve). The inset shows schematic of the resistive switching device architecture. b) Plot of the OFF/ON resistance ratio (right axis) and LRS (ON) current and HRS (OFF) current at a read voltage of 0.2 V as a function of GeO_2 NP concentration. c) and d) Log *I* – log *V* plots of the bipolar RS memory at R_m= 0.05% and 0.15%, respectively.

Successive *I*- *V* curves for a device with Rm = 0.05% with an initial HRS and Rm = 0.15%, showing reproducible *I*- *V* characteristics and DC endurance performance are shown in Figure S8 and Figure S9, respectively.

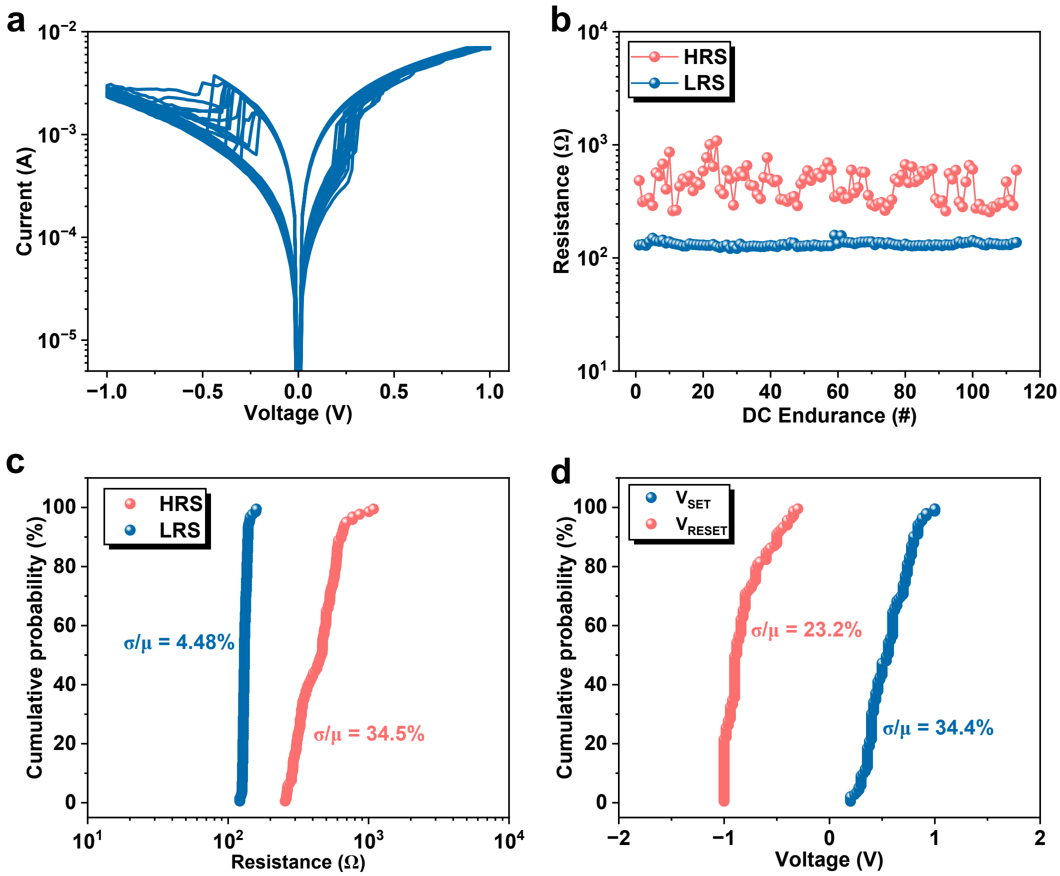

Figure S8. a) Successive *I*-*V* curves of a typical hybrid device, ITO/GeO_2:PMMA/Al (with Rm = 0.05%), exhibiting the HRS state in its pristine condition. b) DC endurance for the HRS and LRS. Cumulative probability of the c) HRS and LRS and d) V_{SET} and V_{RESET}. Although the device is initially in the HRS, the current level for the HRS is still higher (by more than two orders) than that of devices that are LRS in their pristine state. (i.e. the former devices are more conducting because of the presence of conducting filaments). This results in a small window between HRS and LRS of about 4 in comparison with devices that have an LRS in their pristine condition, which instead typically exhibit a larger window of more two orders of magnitude.

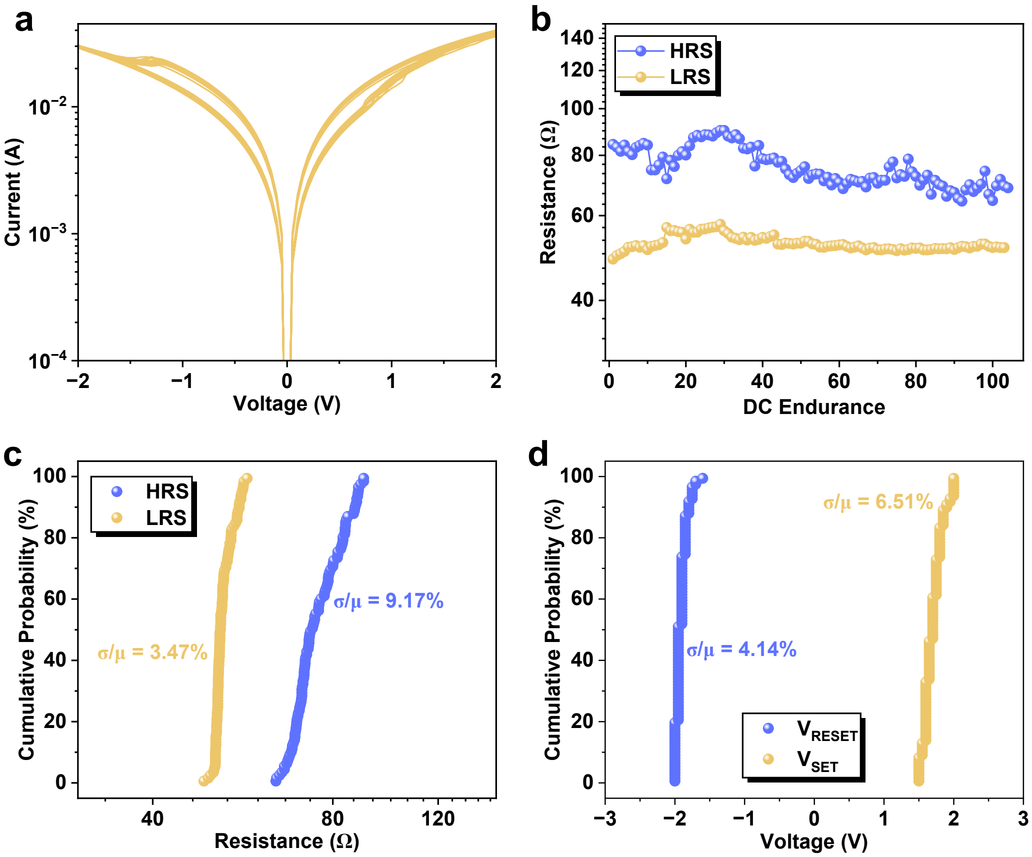

Figure S9. a) Successive *I-V* curves of a typical hybrid device, ITO/GeO_2:PMMA/Al (with Rm = 0.15%), showing reproducible switching performance. b) DC endurance for the HRS and LRS. c) Cumulative probability of the HRS and LRS, and d) V_{SET} and V_{RESET}.

3. Single Layer and Bi-layer GeO_2 NP Devices

Besides mixed hybrid memristor devices, another two device architectures were studied. The first device structure, called a single layer device, consists of only GeO_2 NPs, and has the configuration of ITO/GeO_2/Al. The second device structure, called a bi-layer device, consists of two distinct layers of the GeO_2 NPs and PMMA, and has the configuration of ITO/PMMA/GeO_2/Al. These device architectures can be seen from the insets in Figure S10a.

Both the ITO/GeO_2/Al and ITO/PMMA/GeO_2/Al based devices were fabricated by spin-coating a mixed GeO_2 NPs/toluene solution (800 rpm, 10 s) onto the substrates, followed by an annealing process at 100 °C for an hour to form a thin-film of approximate thickness ~150 – 200 nm. However, in the case of the ITO/PMMA/GeO_2/Al devices, prior to depositing the GeO_2 NPs film, a 40 nm thick of PMMA was deposited by spin-coating a PMMA solution (3000 rpm, 30 s) on the ITO substrate with annealing at 40 °C for 10 minutes. For both device types, a final Al films (200 nm) were thermally deposited onto the substrates using a shadow mask with 400 μm diameter circular dots to act as the top electrodes.

Figure S10a shows a comparison of the *I-V* sweeps of three different device types, the single layer GeO_2 NP device, bi-layer GeO_2 NP/PMMA device and the control device containing only PMMA. The GeO_2 NP containing devices were more conductive than the other devices and were swept at low voltages between –1.3 V and 1.3 V, see Figure S10a (yellow curve). As observed previously in other nanoparticle based resistive switching memory devices[8,9] no forming step was needed to initiate the switching process. The single layer device, ITO/GeO_2/Al, had the lowest resistivity with an initial LRS. Devices of this nature exhibited a bipolar resistive switching behavior (negative SET and positive RESET) with a low OFF/ON resistance ratio of ~ 2. Although the resistance OFF/ON ratio was small, these devices, having very smooth *I-V* sweeps and no sudden jumps in the current, exhibited the most reliable switching properties of all of the GeO_2 device types. The *I-V* sweeps for a device showing a reliable and reproducible switching are presented in Figure S10b.

Figure S10a also shows an *I*-*V* sweep of the control device containing only an insulating layer of PMMA (200 nm), ITO/PMMA/Al, (red curve). This device, as expected, had the highest resistivity because of the highly insulating nature of the PMMA material.[10] The device also showed no resistive switching between HRS and LRS, even though the device was swept to higher voltages, ±2 V.

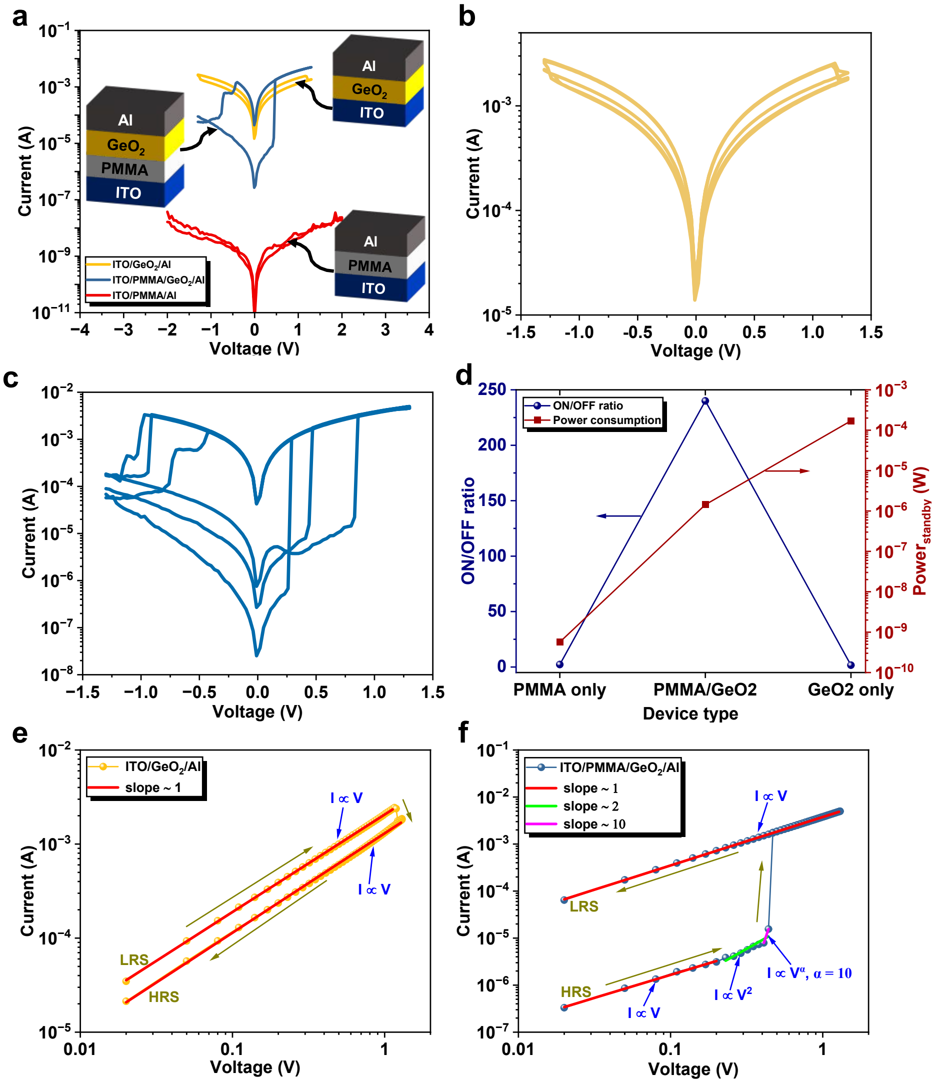

Figure S10. a) *I-V* curves of resistive switching devices based on ITO/GeO_2/Al (yellow curve), ITO/PMMA/GeO_2/Al (blue curve) and ITO/PMMA/Al (red curve). The inset show schematics of the three memristor device architectures. b) Successive *I-V* curves of a single layer ITO/GeO_2/Al device, showing a reproducible switching performance. c) Successive *I-V* curves of a bi-layer ITO/PMMA/GeO_2/Al resistive switching devices. d) Plot of the OFF/ON ratio (left axis) at a read voltage of 0.2 V and power consumption (right axis) at a read voltage of 0.3 V as a function of device type. e) Fits to an ohmic conduction mechanism for the single layer ITO/GeO_2/Al *device.* f) An SCLC conduction mechanism fit for the bi-layer ITO/PMMA/GeO_2/Al device.

The bi-layer device, ITO/PMMA/GeO_2/Al, also exhibited resistive switching effects but had a vastly improved resistance OFF/ON ratio of three orders of magnitude, see Figure S10a (blue curve). Successive *I-V* curves for a device showing a reliable and reproducible switching are presented in Figure S10c. In contrast to the ITO/GeO_2/Al device, which was in the LRS state in its pristine state, the ITO/PMMA/GeO_2/Al device existed first in the HRS. There are also other notable differences between the two device types. The single layer device typically switched at much higher potentials, ~ 1.2 V, in contrast to ~ 0.47 V for the bi-layer device. The type of switching was also very different. In the single layer device the switch between the high and low resistance states occurred smoothly whereas the bi-layer device exhibited very sharp, first-order like transitions, which is typically indicative of a filamentary switching process, resulting in a larger OFF/ON resistance ratio of about 3 orders of magnitude, see Figure S10d. Additionally, adding the PMMA layer to the GeO_2 NPs decreased the power consumption from 171 μW for the single layer device to 1.43 μW for the bi-layer device, as shown in Figure S10d. The power

consumption ($Power_{standby}= I_{HRS}\times V_{read}$) was calculated at a read voltage of 300 mV and the OFF currents of the devices. These comparisons indicate the switching characteristics of inorganic GeO_2 NPs can be improved by the adding of organic PMMA, making the bi-layer device a promising candidate for fabrication low power operation and large OFF/ON resistance ratio non-volatile memories.[11]

To explore the origin of the resistive switching characteristics in the single layer (GeO_2) and bi-layer (PMMA/GeO_2) devices, the conduction mechanisms of the HRS and LRS for both devices were investigated. Figure S10e shows the plot of log I vs. log V for the single layer GeO_2 NP device in the HRS and LRS. Since the device is initially in the LRS it is likely that conductive pathways of some type are already present within the switching material after device fabrication. The straight lines with a slope of approximately 1 for both the HRS and LRS indicate ohmic conduction, which is usually attributed to free carriers being thermally generated.[12] The switch to the HRS at approximately 1.0 V is fast and not much relevant information can be gained from fits in this region. On the other hand, the bi-layer device, whilst exhibiting ohmic conduction for the LRS and for the HRS state at low applied potential, Figure S10f, undergoes a very large jump from high resistance to low resistance at 0.47 V. The shape of this transition has the hallmark of a mechanism involving space charge limited conduction (SCLC), which is a well-known effect that occurs in insulating materials that contain trap states. In this scenario ohmic conduction occurs at low potential, but as the voltage is increased there is a transition to a trap-controlled

SCLC conduction regime ($0.23 \leq V \leq 0.41$), having a characteristic slope of 2.0 on a log-log graph and described by the Mott-Gurney law,[13] as given by:

$$J = \frac{9\varepsilon\mu V^2}{8d^3} \tag{S1}$$

where J is the current density, ε is the dielectric constant, μ is the free carrier mobility, V is the applied voltage, and d is the insulator thickness.

Further increment in the applied voltage up to 0.44 V causes a sharp, first-order transition with a slope of 10. Typically, large values of slope in the trap-filling regime indicates the presence of a large number of traps with an exponential distribution over energy, most likely this injection occurs at the GeO_2 NPs/PMMA interface with the trap states being in the GeO_2 NPs,[12,14,15] since the control PMMA device did not show a RS effect and/or trapping effect. It is also worth mentioning that the trapping effect may occur at the GeO_2 NPs/PMMA interface due to the dangling bonds on the NP surface. After the filling of all traps there should be a change in the conduction mechanism to a trap-free space charge limited conduction (TF-SCLC). However, in this case the slope of the I-V curve is already 1.0, in contrast to the normal slope value of 2.0, which indicates that the TF-SCLC state might be short-lived or more likely, the switch to the LRS state has already occurred as evidenced by the value of the slope being the same as the return path from the maximum voltage (1.3 V) back to 0 V. The current transport in the return path is again governed by ohmic conduction.[16,17]

Although both the single layer and bi-layer GeO_2 NPs devices exhibit clear bipolar RS properties at low applied bias, there are significant differences in their *I-V* characteristics and switching properties. Namely, 1. the pristine devices have different initial states (HRS, LRS); 2. the shape of the *I-V* curves and the abrupt HRS/LRS transition; 3. the value of the resistance OFF/ON ratio. We discuss the different switching properties and conduction mechanisms in the following.

The initial resistance state of pristine single layer devices is the LRS, whereas for the bi-layer devices, it is the HRS. The LRS of the single layer devices is easier to understand since it likely indicates the GeO_2 NPs contain a high amount of oxygen vacancy defects, (V_O),[18] which makes the thin-film more conducting. The GeO_2 NP thin-film is also in direct contact with the Al and ITO electrodes and from the shape and symmetry of the *I-V* curve, it appears that there is a good ohmic contact with the electrodes, facilitating easy charge transport in both the LRS and HRS case. The formation of ohmic contact in both the HRS and LRS in the single layer device is evidenced by the slope of the graph (≈1), symmetric profile of the *I-V* curve and the V_{SET} and V_{RESET}, having the same absolute magnitude i.e. −1.26 V and +1.22 V, respectively. The ohmic contacts appear to be largely unaffected by the bias, but it could be minor changes in this which cause the observed changes in resistance between HRS and LRS.

In the bi-layer devices case, the initial HRS can be attributed to the presence of the 40 nm thick PMMA, which acts as an insulating barrier between the GeO_2 NPs film and the ITO bottom electrode. However, the mechanism of switching is unclear since either SCLC with trap-filling occurs at the GeO_2 NPs/PMMA interface, as shown by the modelling in Figure S10f, or, with the

application of a sufficient potential, oxygen vacancies could migrate from the GeO_2 NPs into the PMMA layer to form a conducting filament,[19] which can produce similar switching characteristics in the *I-V* curve. The thickness of the PMMA layer is quite thin, ~40 nm, and it is quite likely that a few spots exist in the device where it may be substantially thinner. In these places the electric field is larger and filament formation would be easier. This would lead to current hot spots in the devices, which are commonly seen in the field.[20,21] We exclude the migration of aluminum ions into the PMMA in this case since the control device ITO/PMMA/Al exhibited no evidence of switching.

A noticeable difference between the bi-layer device and the single layer device is that the transition from the HRS to the LRS (and vice versa) is smooth for the single layer device. The *I-V* sweeps do not contain abrupt transitions and the shape of sweeps do not significantly change after repeated cycles, indicating the devices are much more stable. This type of resistive switching, often termed homogeneous switching[22] or non-filamentary,[8,23] is typically due to gradual changes in the materials properties or electronic structure of the device, such as changes in the Schottky-barrier heights at interfaces between the functional material and electrode. In contrast, the bi-layer device exhibits sharp, 1st order like transitions between the ON and OFF states with large changes in the conductance, often more than two orders of magnitude. This is typical of the trapping and de-trapping effect-based switching mechanism.[24,25]

The switching mechanism of the bi-layer device is expected to be based on trapping and de-trapping effect that can occur at the PMMA/GeO_2 interface. Figure S11 shows a schematic

diagram of the electronic structure for the ITO/PMMA/GeO_2/Al under a positive voltage polarity, where the ITO is positive with respect to Al. The GeO_2 NPs can act as charge trapping centers at the interface. When a positive voltage is applied, the injected charges are trapped by GeO_2 NP trapping sites and once a sufficient positive voltage is reached, all the trap sites are filled, and as a result switching the device to the LRS. When a negative voltage is applied, the trapped charges are removed, and consequently switching the device to the HRS. This is consistent with the fitting *I-V* curve in Figure S10f, which shows the trap-mediated SCLC effect.

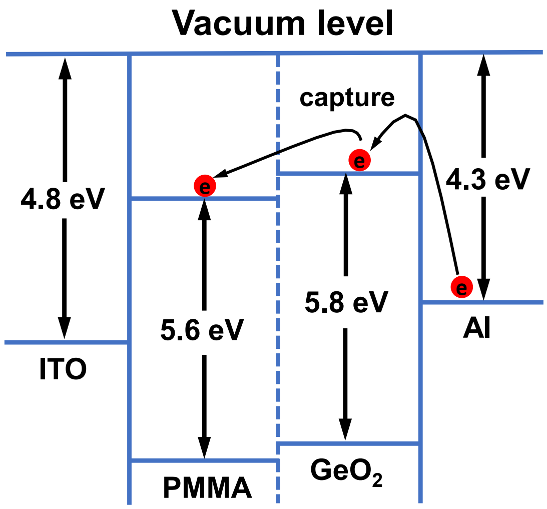

Figure S11. Schematic diagram of the energy levels for the bilayer ITO/PMMA/GeO_2/Al device. The LUMO and HOMO represent the energy levels of the lowest unoccupied molecular orbital and the highest occupied molecular orbital of the PMMA film, respectively. The energy levels for GeO_2 NPs were taken from ref.[26] and those for PMMA from ref.[13] Upon application of a positive voltage on the ITO electrode, electrons are initially injected from the Al electrode into the GeO_2 NPs *via* thermally generated carriers. Increasing the applied voltage gradually increases the

injection process *via* the SCLC, but in this case, injected electrons begin to be trapped by the GeO_2 NP trapping sites. Below the SET voltage, the device is still in the OFF state since some of the trapped electrons are transferred from the GeO_2 NPs to the ITO electrode *via* the LUMO level of PMMA, leaving the GeO_2 NPs partially occupied. Once the GeO_2 NP trap sites are fully occupied, as shown by the sharp increase in current, the device switches to the ON state at a SET voltage of 0.5 V. However, when the voltage is switched to negative bias conditions, the trapped electrons are gradually released, resulting in switching the device to the OFF state at a RESET voltage of ~−1 V.

Regardless of the precise switching mechanism, our research confirms that the addition of a 40 nm thick PMMA layer improves the RS properties of devices by increasing the resistance OFF/ON ratio of devices and decreasing the power consumption. Additionally, the devices are also forming-free and device yield is improved, most likely because the PMMA layer prevents possible short-circuits between the two electrodes during fabrication and device operation.

REFERENCES

(1) Scott, J. F. Raman Spectra of Geo. *Phys Rev B* **1970**, *1* (8).

(2) Gillet, P.; Le Cleac'h, A.; Madon, M. High-Temperature Raman Spectroscopy of SiO2 and GeO2 Polymorphs: Anharmonicity and Thermodynamic Properties at High-Temperatures. *J Geophys Res* **1990**, *95* (B13). https://doi.org/10.1029/jb095ib13p21635.

(3) Tanaka, K.; Yamaguchi, M. Resonant Raman Scattering in GeS 2. *J Non Cryst Solids* **1998**, *227* (230), 757–760.

(4) Bokova, M.; Paraskiva, A.; Fontanari, D.; Cuisset, A.; Kassem, M.; Bychkov, E. Raman Spectroscopy and DFT Modelling of Tl2S-GeS2 Crystals and Glasses. *J Non Cryst Solids* **2023**, *601*. https://doi.org/10.1016/j.jnoncrysol.2022.122055.

(5) Thanh Dao, T.; Viet Tran, T.; Higashimine, K.; Okada, H.; Mott, D.; Maenosono, S.; Murata, H. High-Performance Nonvolatile Write-Once-Read-Many-Times Memory Devices with ZnO Nanoparticles Embedded in Polymethylmethacrylate. *Appl Phys Lett* **2011**, *99* (23), 2009–2012. https://doi.org/10.1063/1.3665937.

(6) Kim, W.; Rhee, S. Effect of the Top Electrode Material on the Resistive Switching of TiO 2 Thin Film. *Microelectron Eng* **2010**, *87* (2), 98–103. https://doi.org/10.1016/j.mee.2009.05.023.

(7) Peng, H. Y.; Pu, L.; Wu, J. C.; Cha, D.; Hong, J. H.; Lin, W. N.; Li, Y. Y.; Ding, J. F.; David, A.; Li, K.; Wu, T. Effects of Electrode Material and Configuration on the Characteristics of Planar Resistive Switching Devices Effects of Electrode Material and Configuration on the Characteristics of Planar Resistive Switching Devices. *APL Mater* **2013**, *052106*, 1–7. https://doi.org/10.1063/1.4827597.

(8) Jaafar, A. H.; Gee, A.; Kemp, N. T. Nanorods vs Nanoparticles: A Comparison Study of Au/ZnO-PMMA/Au Non-Volatile Memory Devices Showing the Importance of Nanostructure Geometry on Conduction Mechanisms and Switching Properties. *IEEE Trans Nanotechnol* **2020**, *19*, 236–246. https://doi.org/10.1109/TNANO.2019.2949759.

(9) Jaafar, A. H.; Neill, M. O.; Kelly, S. M.; Verrelli, E.; Kemp, N. T. Percolation Threshold Enables Optical Resistive-Memory Switching and Light-Tuneable Synaptic Learning in Segregated Nanocomposites. *Adv Electron Mater* **2019**, *5* (1900197), 1–7. https://doi.org/10.1002/aelm.201900197.

(10) Huang, T.-S.; Su, Y.-K.; Wang, P.-C. Study of Organic Thin Film Transistor with Polymethylmethacrylate as a Dielectric Layer. *Appl Phys Lett* **2007**, *91* (9), 092116. https://doi.org/10.1063/1.2775333.

(11) Jaafar, A. H.; Meng, L.; Zhang, T.; Guo, D.; Newbrook, D.; Zhang, W.; Reid, G.; de Groot, C. H. (Kees); Bartlett, P. N.; Huang, R. Flexible Memristor Devices Using Hybrid Polymer/Electrodeposited GeSbTe Nanoscale Thin Films. *ACS Appl Nano Mater 5* (12), 17711–17720. https://doi.org/10.1021/acsanm.2c03639.

(12) Son, D. I.; Kim, T. W.; Shim, J. H.; Jung, J. H.; Lee, D. U.; Lee, J. M.; Park, W. il; Choi, W. K. Flexible Organic Bistable Devices Based on Graphene Embedded in an Insulating Poly(Methyl Methacrylate) Polymer Layer. *Nano Lett* **2010**, *10* (7), 2441–2447. https://doi.org/10.1021/nl1006036.

(13) Chiu, F. A Review on Conduction Mechanisms in Dielectric Films. *Advances in Materials Science and Engineering* **2014**, *ID 578168*, 18. https://doi.org/10.1155/2014/578168.

(14) Tu, C. H.; Lai, Y. S.; Kwong, D. L. Memory Effect in the Current - Voltage Characteristic of 8-Hydroquinoline Aluminum Salt Films. *IEEE Electron Device Letters* **2006**, *27* (5), 354–356. https://doi.org/10.1109/LED.2006.872915.

(15) Son, D.; Park, D.; Kim, J. Bistable Organic Memory Device with Gold Nanoparticles Embedded in a Conducting Poly (N-Vinylcarbazole) Colloids Hybrid. *The Journal of Physical Chemistry C* **2010**, *115*, 2341–2348.

(16) Du, G.; Wang, C.; Li, H.; Mao, Q.; Ji, Z.; Du, G.; Wang, C.; Li, H.; Mao, Q.; Ji, Z. Bidirectional Threshold Switching Characteristics in Ag / ZrO2 / Pt Electrochemical Metallization Cells. *AIP Adv* **2016**, *6* (085316), 1–6. https://doi.org/10.1063/1.4961709.

(17) Yang, Y. C.; Pan, F.; Liu, Q.; Liu, M.; Zeng, F. Fully Room-Temperature-Fabricated Nonvolatile Resistive Memory for Ultrafast and High-Density Memory Application. *Nano Lett* **2009**, *9* (4), 1636–1643.

(18) Shaposhnikov, A. V.; Perevalov, T. V.; Gritsenko, V. A.; Cheng, C. H.; Chin, A. Mechanism of GeO 2 Resistive Switching Based on the Multi-Phonon Assisted Tunneling between Traps. *Appl Phys Lett* **2012**, *100* (24). https://doi.org/10.1063/1.4729589.

(19) Liu, H.; Dong, Y.; Galib, M.; Cai, Z.; Stan, L.; Zhang, L.; Suwardi, A.; Wu, J.; Cao, J.; Tan, C. K. I.; Sankaranarayanan, S. K. R. S.; Narayanan, B.; Zhou, H.; Fong, D. D. Controlled Formation of Conduction Channels in Memristive Devices Observed by X-Ray

Multimodal Imaging. *Advanced Materials* **2022**, *34* (35). https://doi.org/10.1002/adma.202203209.

(20) Yasaei, P.; Murthy, A. A.; Xu, Y.; dos Reis, R.; Shekhawat, G. S.; Dravid, V. P. Spatial Mapping of Hot-Spots at Lateral Heterogeneities in Monolayer Transition Metal Dichalcogenides. *Advanced Materials* **2019**, *31* (24). https://doi.org/10.1002/adma.201808244.

(21) Deshmukh, S.; Muñoz Rojo, M.; Yalon, E.; Vaziri, S.; Koroglu, C.; Islam, R.; Iglesias, R. A.; Saraswat, K.; Pop, E. Direct Measurement of Nanoscale Filamentary Hot Spots in Resistive Memory Devices. *Sci Adv* **2022**, *8*, 1–7.

(22) Huang, C. H.; Huang, J. S.; Lai, C. C.; Huang, H. W.; Lin, S. J.; Chueh, Y. L. Manipulated Transformation of Filamentary and Homogeneous Resistive Switching on ZnO Thin Film Memristor with Controllable Multistate. *ACS Appl Mater Interfaces* **2013**, *5* (13), 6017–6023. https://doi.org/10.1021/am4007287.

(23) Jaafar, A. H.; Gray, R. J.; Verrelli, E.; O'Neill, M.; Kelly, Stephen. M.; Kemp, N. T. Reversible Optical Switching Memristors with Tunable STDP Synaptic Plasticity: A Route to Hierarchical Control in Artificial Intelligent Systems. *Nanoscale* **2017**, *9* (43), 17091–17098. https://doi.org/10.1039/C7NR06138B.

(24) Quynh, N. P. L. P.; Thi, T. U. D.; Tran, K. M.; Vu, H. N.; Ta, H. K. T.; Tran, C. V.; Phan, T. B.; Pham, N. K. Improving Memory Performance of PVA:ZnO

Nanocomposite: The Experimental and Theoretical Approaches. *Appl Surf Sci* **2021**, *537*. https://doi.org/10.1016/j.apsusc.2020.148000.

(25) Bera, J.; Betal, A.; Sharma, A.; Shankar, U.; Rath, A. K.; Sahu, S. CdSe Quantum Dot-Based Nanocomposites for Ultralow-Power Memristors. *ACS Appl Nano Mater* **2022**, *5* (6), 8502–8510. https://doi.org/10.1021/acsanm.2c01894.

(26) Fadida, S.; Eizenberg, M.; Nyns, L.; Van Elshocht, S.; Caymax, M. Band Alignment of Hf-Zr Oxides on Al2O3/GeO 2/Ge Stacks. *Microelectron Eng* **2011**, *88* (7), 1557–1559. https://doi.org/10.1016/j.mee.2011.03.075.
